# Supplementary material for: Synthesizing Complex-Valued Multicoil MRI Data from Magnitude-Only Images
Source: Bioengineering (Basel). 2023 Mar 14;10(3):358. doi: 10.3390/bioengineering10030358 (PMC10045391; doi:10.3390/bioengineering10030358)
Supplement: Supplementary file 1 [file bioengineering-10-00358-s001.zip › bioengineering-2225976-supplementary.pdf]

Article

Synthesizing Complex-Valued Multicoil MRI Data from Magnitude-only Images

Nikhil Deveshwar [1,2,3], Abhejit Rajagopal [2], Sule Sahin [1,2], Efrat Shimron [3] and Peder E. Z. Larson [1,2*]

1 UC Berkeley-UCSF Graduate Program ing Bioengineering, Berkeley and San Francisco, CA, USA
2 Department of Radiology and Biomedical Imaging, University of California San Francisco, San Francisco, CA, USA, USA

3 Department of Electrical Engineering and Computer Sciences, University of California, Berkeley, Berkeley, CA
* Correspondence: peder.larson@ucsf.edu

Table S1. PSNR values for VarNet trained on different types of phase at various acceleration factors for the 16-coil dataset. Bold values indicate the best performing type of phase (not including ground truth).

Phase type	R=4	R=6	R=8	R=10
Ground truth	32.915	29.995	28.4529	27.692
Synthetic Phase (ours)	**31.196**	**28.602**	**28.039**	**26.378**
Sinusoidal Phase	23.652	23.229	22.99	22.590
Random Phase	23.938	22.255	22.028	22.581
Zero Phase	23.911	23.589	22.976	21.518

Table S2. PSNR values for VarNet trained on different types of phase at various acceleration factors for the 20-coil dataset. Bold values indicate the best performing type of phase (not including ground truth).

Phase Type	R=4	R=6	R=8	R=10
Ground Truth	32.605	30.0454	28.367	27.747
Synthetic Phase(ours)	**30.484**	**29.712**	**27.054**	**26.314**
Sinusoidal Phase	23.068	22.576	22.255	22.020
Random Phase	22.994	22.771	22.434	22.066
Zero Phase	2.947	22.74	22.388	21.826

Table S3. NMSE values for VarNet trained on different types of phase at various acceleration factors for the 16-coil dataset. Bold values indicate the best performing type of phase (not including ground truth).

Phase Type	R=4	R=6	R=8	R=10
Ground Truth	0.0133	0.0213	0.029295	0.03615
Synthetic Phase (ours)	**0.0195**	**0.0309**	**0.0336**	**0.043**
Sinusoidal Phase	0.096	0.099	0.108	0.115
Random Phase	0.093	0.102	0.103	0.116
Zero Phase	0.092	0.101	0.107	0.152

Table S4. NMSE values for VarNet trained on different types of phase at various acceleration factors for the 20-coil dataset. Bold values indicate the best performing type of phase (not including ground truth).

Phase Type	R=4	R=6	R=8	R=10
Ground Truth	0.0176	0.0244	0.0354	0.0383
Synthetic Phase (ours)	**0.0265**	**0.0337**	**0.0520**	**0.0613**
Sinusoidal Phase	0.249	0.253	0.263	0.304
Random Phase	0.247	0.2409	0.251	0.284
Zero Phase	0.266	0.230	0.243	0.301

Table S5. SSIM values for VarNet trained on different types of phase at various acceleration factors for the 16-coil dataset. Bold values indicate the best performing type of phase (not including ground truth).

Phase Type	R=4	R=6	R=8	R=10
Ground Truth	0.8425	0.816	0.773	0.767
Synthetic Phase (ours)	0.7468	**0.745**	0.6889	0.6511
Sinusoidal Phase	**0.768**	0.736	**0.721**	**0.694**
Random Phase	0.668	0.639	0.618	0.598
Zero Phase	0.668	0.645	0.617	0.546

Table S6. SSIM values for VarNet trained on different types of phase at various acceleration factors for the 20-coil dataset. Bold values indicate the best performing type of phase (not including ground truth).

Phase Type	R=4	R=6	R=8	R=10
Ground Truth	0.8189	0.790	0.731	0.722
Synthetic Phase (ours)	**0.819**	**0.784**	**0.681**	**0.671**
Sinusoidal Phase	0.701	0.687	0.674	0.655
Random Phase	0.708	0.687	0.669	0.652
Zero Phase	0.709	0.691	0.672	0.644
